# Use of Biometrics for Records Deduplication: Case Study of the National Data Repository in Nigeria

**DOI:** 10.2196/67580

**Published:** 2025-08-26

**Authors:** Ademola Oladipo, Ibrahim Dalhatu, Stephen Taiye Balogun, Moyosola Bamidele, Ayodele Fagbemi, Isah Ahmed Abbas, Nannim Nalda, Richard Ugbena, Jude Orjih, Timothy A Efuntoye, Brooke Doman, Sadhna Patel, Herman Tolentino, Daniel Rosen, James Kariuki, Johnson Alonge, Kehinde Balogun, Nnamdi Umeh, Gibril Gomez, Oludare Onimode, Olaposi Olatoregun, Jay Osi Samuels, Adebobola Bashorun

**Affiliations:** 1 Division of Global HIV and TB, US Centers for Disease Control and Prevention, 1600 Clifton Road NE, Atlanta, GA, 30329, United States, 1 404 218 2662; 2 Division of Global HIV and TB, US Centers for Disease Control and Prevention, Abuja, Nigeria; 3 Public Health Information, Surveillance Solutions and Systems Project, Abuja, Nigeria; 4 National AIDS and STI Control Program (NASCP), Ministry of Health, Abuja, Nigeria

**Keywords:** unique client identification, fingerprint, deduplication, data quality, PEPFAR-CDC, Nigeria

## Abstract

**Background:**

Nigeria has made significant investments in client-level electronic health systems, including the Nigeria Medical Record System (NMRS) and the National Data Repository (NDR), with funding from the US President’s Emergency Plan for AIDS Relief through the US Centers for Disease Control and Prevention (US CDC). A biometric system was used across the US CDC–supported program in Nigeria to consistently track and monitor service uptake by people living with HIV during this period. The system was used to conduct deduplication analysis with the goal of preventing double counting and improving data integrity across all the US CDC-supported treatment sites (health facilities and community sites).

**Objective:**

We describe the fingerprint biometric system in Nigeria and the process used for deduplicating health records of people living with HIV, including preliminary results.

**Methods:**

The fingerprint biometric system leveraged the availability of the electronic NMRS at health facilities and the NDR. The integration of the fingerprint biometric module into the NMRS enabled fingerprints capture using SecuGen devices. Stakeholder engagement and capacity building were conducted with people living with HIV and health facility staff for fingerprint capture, storage, and transmission of the fingerprint templates to the NDR. Deduplication of the fingerprint templates was conducted in the automated biometric information system that is integrated with the NDR.

**Results:**

We implemented fingerprint capture for 1,538,971 people living with HIV to deduplicate records from 1,141 treatment sites to improve the reliability and uniqueness of the system of records. Preliminary data showed that of the 1,538,971 records assessed by 30th June 2024, 1,520,187 of the active records (98.78%) had valid fingerprints, and 1,264,299 (83.17%) of the records with valid fingerprints were unique.

**Conclusions:**

The implementation of a biometric system using fingerprint data allowed the identification of potentially duplicate records for resolution, thereby improving the quality of HIV treatment data for HIV program planning.

## Introduction

**Figure 1.**
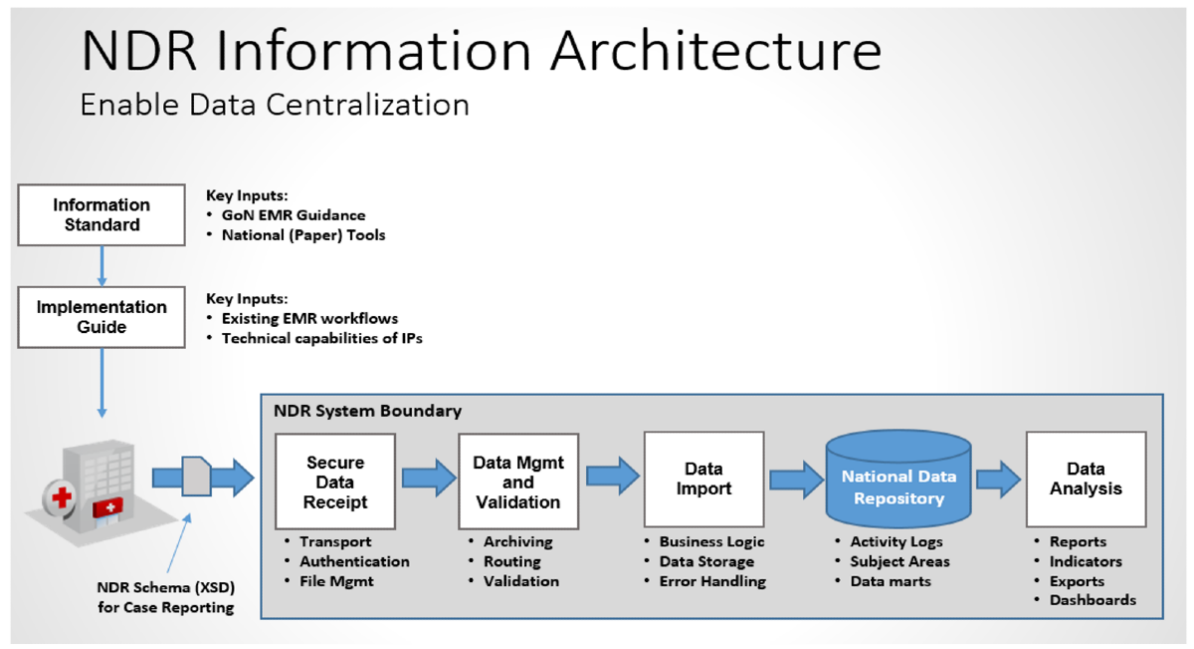
The logical architecture of the National Data Repository (EMR: electronic medical record; GoN: Goverment of Nigeria; IP: implementing partners; Mgmt: management; NDR: National Data Repository; XSD: XML Schema Definition).

Nigeria has made significant investments in client-level electronic health systems, including the Nigeria Medical Record System (NMRS) and the National Data Repository (NDR), with funding from the US President’s Emergency Plan for AIDS Relief (PEPFAR) through the US Centers for Disease Control and Prevention (US CDC) [1]. The NDR is a centralized repository of client-level data from disease programs of public health importance in Nigeria, starting with data from the HIV/AIDS program. The data from all HIV/AIDS-specific electronic medical records (EMRs) in Nigeria are transported and received securely using Secure Shell File Transfer Protocol, validated, and imported into the NDR. Figure 1 shows the high-level representation of the logical architecture of the NDR. The NDR data dictionary, information exchange standard, and case report message enable data exchange. The case-based message approach allows for the upload of only encounters that have not been pre-reported; thus, clients’ records can be updated on the NDR and not replaced with each upload. Each message file submitted to the NDR has the data of a client that strictly follows the NDR information exchange standard. Data are XML-encoded in a hierarchical structure.

The NMRS is an electronic medical record system that was built on the OpenMRS software (Public Health Information, Surveillance Solutions and Systems Project, APIN Public Health Initiative), and it is used across 1141 health facilities and community sites in 19 states of Nigeria to manage records for people living with HIV supported by the PEPFAR program through the US CDC [1]. Community sites are unconventional locations outside of but linked to the health system, which are managed by community-based organizations (CBOs). The NMRS helps to improve the efficiency of collecting, managing, and reporting health information of people living with HIV [1-4]. Service delivery documentation takes place initially in the paper-based records; however, they are transferred by data entry clerks into the NMRS within 24 hours of documentation [1]. Records in the NMRS help health facilities to identify and verify clients when they receive services. The NMRS provides a comprehensive system for the availability and use of digital health records for decision-making.

In US CDC-supported facilities, the NMRS was leveraged to implement the fingerprint capture initiative. The fingerprint is a widely acceptable form of biometric data that provides more definitive client identification [5-7]. Its use in identification is based on the science that no 2 individuals have the same fingerprint and that fingerprints remain consistent over a lifetime. The orientation of the ridges and valleys of each finger provides the distinction between any 2 individuals [6,7]. The uniqueness of fingerprints can be enhanced by the shape, number, and arrangement of the fingerprint patterns [6,7]. Stored fingerprint templates can enable client identification, verification, and authentication at the point of service, ensuring the provision of safe, secure, and effective care to the right client. This can improve the client treatment experience, as they can receive antiretroviral therapy services at any health facility of their choice and have their health records retrieved via the Patient Identification Management System [6-8]. Additionally, it assists in accounting for service linkage within and between sites and fewer medication errors due to misidentification.

People living with HIV are assigned hospital numbers and hand cards. These hand cards have composite identifiers, including hospital name, people living with HIV’s name, hospital number, date of birth, and appointment schedule, which help to identify people living with HIV at the point of service delivery. However, the hand cards and hospital numbers are not universal across sites. As a result, when people living with HIV moved across sites or misplaced their hand cards, it was difficult to identify them, and new records might be created. Given this unique identification challenge, and to improve data quality and identification of unique records across all the 1,141 sites, the Government of Nigeria (GoN), in collaboration with the PEPFAR program, introduced fingerprint capture to be associated with individual medical records to address the problem. Stored fingerprint templates transported and received in the NDR are used for matching and deduplicating records to give account of the unique clients receiving treatment.

We present preliminary results and associated insights from the client records deduplication process we conducted using fingerprint data in the NDR.

## Methods

### Ethical Considerations

This activity was reviewed by the US CDC, deemed not research, and was conducted consistent with applicable federal law and the US CDC policy. The requirements for written informed consent were waived as the preliminary analysis was summarized data by the NDR. Investigators did not interact with human subjects or access personally identifiable information.

### Biometric Module in National Medical Records System

**Figure 2.**
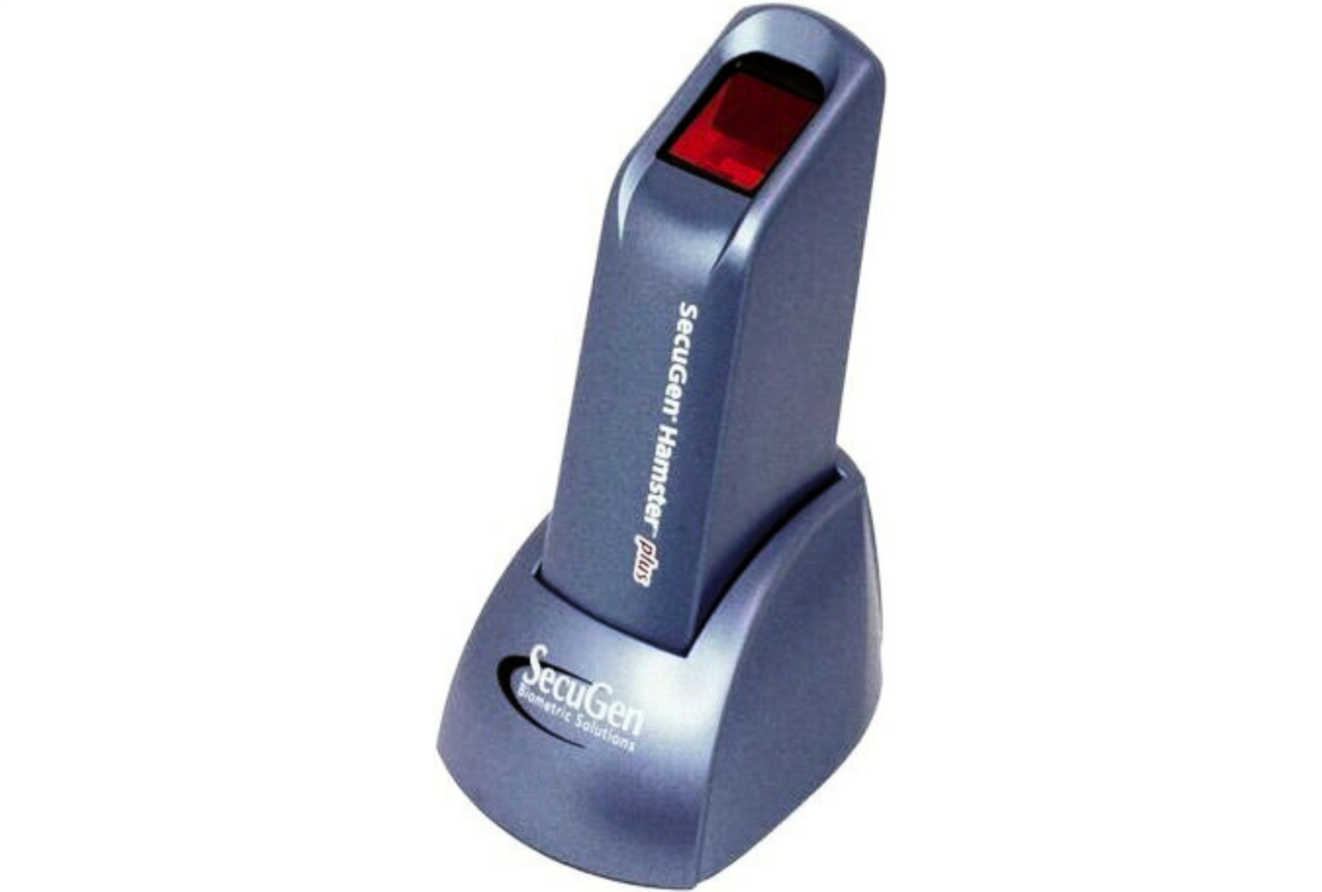
SecuGen Hamster Plus fingerprint scanner.

A biometrics module was integrated into the NMRS in 2019 to allow for fingerprint capture at the service delivery points. Fingerprints were captured with an optical technology, the SecuGen fingerprint scanner [9]. SecuGen has a range of features for individual users, software development kits (SDKs), and other related applications such as access control, time and attendance tracking, and identity verification [9,10]. The SecuGen Hamster Plus (Figure 2) was chosen as the preferred optical sensor due to its popularity, versatility, and advanced features, using the patented SEIR fingerprint biometric technology [9,11-14].

SecuGen offers a diverse range of fingerprint readers available in different forms, including desktop, handheld, and mobile devices [15]. These readers are designed to capture high-quality fingerprint images rapidly and accurately, making them ideal for deployment in high-traffic environments where speed and precision are crucial. The core technology driving SecuGen’s capabilities lies in its proprietary SEIR fingerprint recognition algorithm, developed to provide precise and reliable fingerprint recognition in a wide array of scenarios [15]. This algorithm works in conjunction with high-quality fingerprint sensors to capture and analyze fingerprint data, comparing it against a database of previously registered fingerprints to authenticate users.

The biometric modules in both the enterprise and point of care NMRS were developed using SecuGen’s SDK. This connects the SecuGen fingerprint reader to the computers and mobile phones. Fingerprints are stored in the NMRS while simultaneously linking them to the corresponding client records for which they were captured. By leveraging the functionality provided by the SecuGen SDK, the biometric module facilitated the seamless integration of fingerprint capture and storage within the NMRS.

### Fingerprint Capture process

Fingerprint capture was integrated into the service delivery flow at the facility or community site. To ensure consistency of the fingerprint capture and reporting process, standard operating procedures were developed to guide health facilities, CBOs, and implementing partners to follow and conform to the recommended process.

To completely collect fingerprint data for all people living with HIV 15 years and older. Fingerprints for all new people living with HIV starting ART were captured during the initial enrollment at the medical records room as part of registration or triaging before clinical consultation. People living with HIV already receiving ART and returning to continue care were captured during medication refills or blood draw visits. Once valid fingerprints were collected for people living with HIV, they were not collected again at subsequent visits. This process was adapted for clients receiving treatment services in the community. The NMRS with the biometric module was installed on Android-based mobile phones. At the beginning of each week, community-based volunteers report to the health facility or CBO site they work for with the mobile phones to have the records of clients planned for services during the week preloaded. As services are provided in the field, fingerprints are captured for new and returning clients. At the end of the same week, the volunteers return to the site where records of services, including fingerprints collected, are synchronized with client records in the site’s database.

To ensure comprehensive identification, we encouraged the capture of all fingerprints from all clients, with a minimum requirement of at least 6 fingers without repeating the capture of any finger. During the capturing process, the SecuGen device assessed the quality of each fingerprint captured. If the quality of a particular fingerprint was below 60%, the system flagged it for immediate repeat capture of that specific finger to ensure accurate and reliable data [11,16]. The biometric module was designed to indicate the quality of fingerprint capture. In addition, the biometric module had the capability to identify, flag, and disable fingerprint capture for clients that had valid fingerprints stored in the NMRS.

The captured fingerprints for each client were stored as templates and linked to the client’s medical record and the unique identifier generated by the NMRS. As part of routine client record extraction and reporting, the captured fingerprint templates were automatically extracted along with the corresponding client record in an Extensible Markup Language file. This file was then reported to the NDR alongside other service delivery data. Within the NDR, the captured fingerprint templates were stored in the biometric table of the NDR database. The NDR implemented validation checks to ensure that all records in the biometric data table contained valid prints, that is, meeting the required minimum number of fingers and quality of capture.

### NDR-ABIS Integration and Deduplication

**Figure 3.**
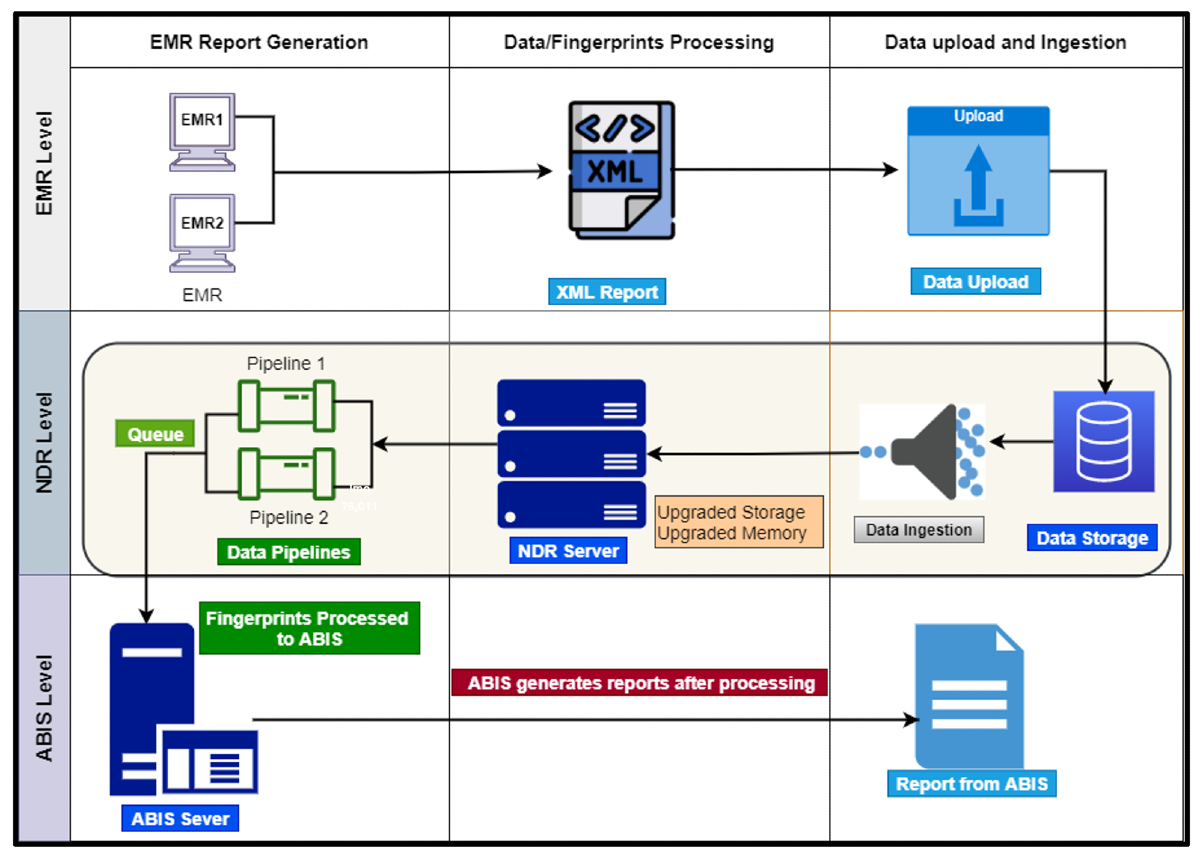
A high-level fingerprint flow and connection between the Nigeria Medical Record System, National Data Repository (NDR), and automated biometric identification system (ABIS). EMR: electronic medical record.

An automated biometric identification system (ABIS) is a technological solution that uses biometric data to identify individuals [17-19]. ABIS MegaMatcher was designed to automate the process of matching and verifying biometric traits, such as fingerprints, against a database of pre-registered biometric records [18,19]. To enable fingerprint template matching and verification, the NDR established a bi-directional connection with the ABIS MegaMatcher. The NDR transmitted captured fingerprint templates to the ABIS MegaMatcher, which performed the matching process against other fingerprint templates in its database. The schematic diagram in Figure 3 illustrates an architectural diagram of the connection between the NMRS, NDR, and ABIS.

The ABIS MegaMatcher offers 2 deployment options: on-premises and cloud service [17]. In Nigeria, it was deployed as a cloud service to enhance accessibility.

The biometric matching process in the ABIS MegaMatcher involved comparing fingerprint templates from one client with fingerprints from all clients stored in the database, following a one-to-all matching approach for verification [17-19]. To ensure fast and accurate matching, the system used proprietary algorithms designed for high-performance biometric processing. These algorithms had the capability of performing up to 1.2 billion comparisons per second on each node of the system, thereby minimizing processing time and increasing accuracy. The outcome of matches in ABIS MegaMatcher is presented in Table 1.

**Table 1.** Outcome of matches in MegaMatcher ABIS (automated biometric identification system) using the Nigeria NDR (National Data Repository) fingerprint data as of 2023.

Outcome	Description
Enrolled	If the fingerprint sample does not match any enrolled templates in the database, the system declares the status as “Ok.” This means that the provided fingerprint data are unique and do not correspond to any registered identity in the system.
Adjudication awaiting	In some cases, when performing a search across a large database, the system may return a list of potential matches or top candidates with a status of adjudication awaiting. These are individuals whose fingerprint data closely resemble the provided sample but may require further verification or human review to determine the exact identity.
Duplicate	When a fingerprint provided by an individual matches an enrolled template in the database, the system declares a match with the status duplicate found. This indicates that the individual’s fingerprint data correspond to a previously registered identity.
Exception	This includes fingerprints affected by system configuration and calibration issues. Other reasons include technical errors or malfunctions such as system communication issues or hardware malfunctions during the transmission of the biometric status result.
No valid data	This is a status returned by NDR when the biometric data (fingerprint) does not adhere to NDR ISO standards or when there are incomplete or missing biometric features, or corrupt data from the electronic medical record.

### Data Security

Rather than storing fingerprints as images, they were converted to templates and encrypted into Base64 hash before they were uploaded on the NDR. The fingerprint template is a condensed, more compact representation of a fingerprint, derived from the raw fingerprint vector data through feature extraction and template generation algorithms [20].

Encrypted fingerprint templates were pushed to ABIS, where they were decrypted and matched to identify duplicates. A role-based access control ensured that users without privileges cannot execute a particular operation.

Duplicate fingerprints were matched by sex and date of birth outside of ABIS to reclassify duplicates as perfect and imperfect duplicates. Imperfect duplicates will be treated like the outcome, “Adjudication awaiting,” which may require further verification or additional human review to arrive at a resolution.

## Results

**Figure 4.**
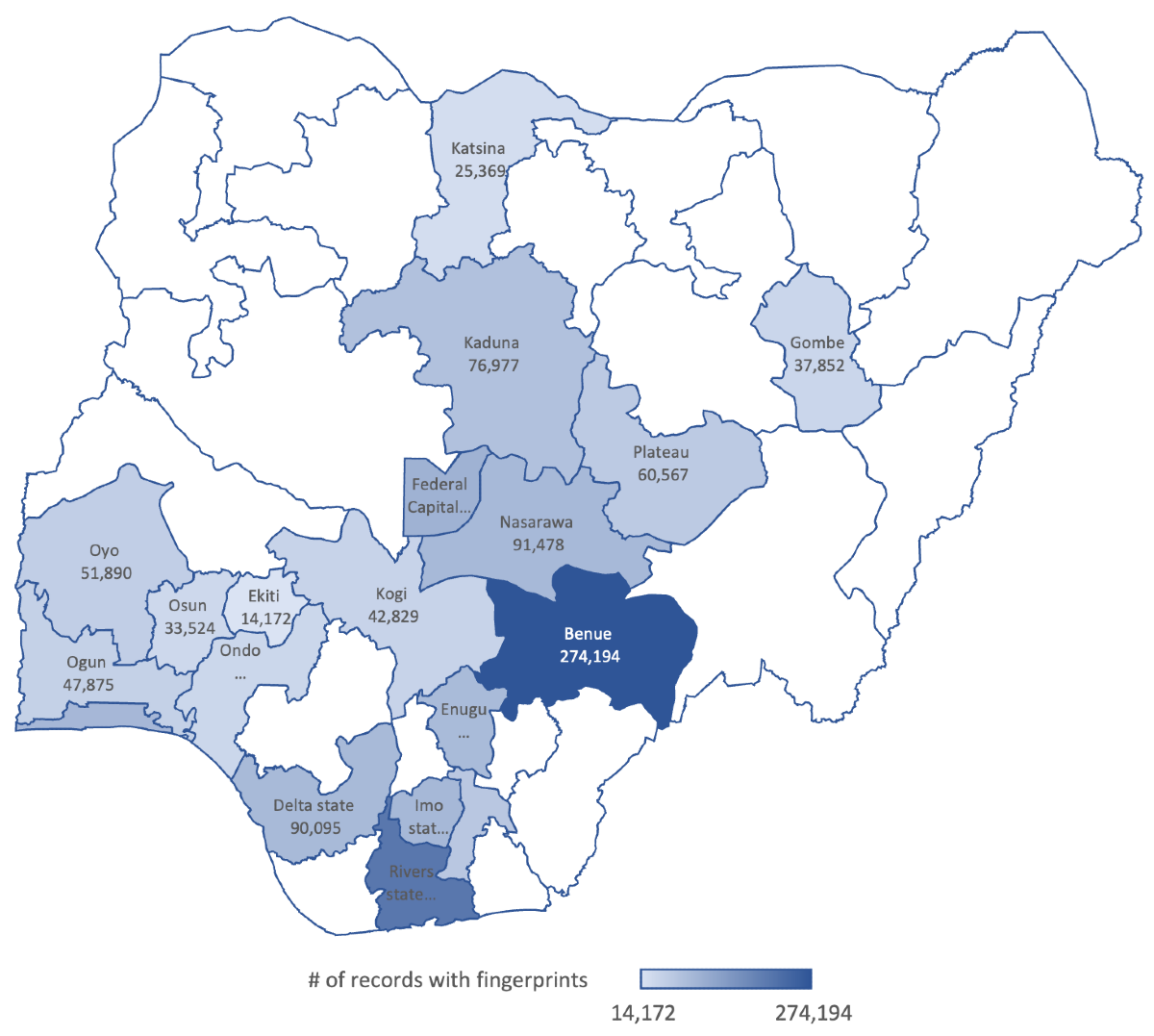
Distribution of fingerprints captured from people living with HIV in the Nigeria National Data Repository by State by June 2024 in CDC-supported states. CDC: Centers for Disease Control and Prevention.

From 2021 through 2024, fingerprints were captured from 1,538,971 people living with HIV aged 15 years and older across 1141 health and community sites supported in 19 states (see Figure 4) by the US CDC in Nigeria. This represents 62% of the 2,483,916 fingerprint records captured across all 36 states and the Federal Capital Territory in Nigeria.

Individual fingerprint templates were domiciled in the respective facility NMRS and reported to the NDR. Out of the 1,538,971 people living with HIV whose fingerprint records were collected, 1,520,187 (98.78%) had valid fingerprints (see Table 2). A valid fingerprint is a status returned by NDR when the biometric data (fingerprint) adhere to NDR ISO Standards and matching can be processed in the ABIS MegaMatcher.

Of the 1,520,187 valid fingerprint data assessed, 1,264,299 (83.17%) of the client records were authenticated as “enrolled,” while 162,120 (10.66%) were determined as “duplicate,” and 93,706 (6.16%) were awaiting adjudication, ie, the ABIS could not conclusively determine if these records should be authenticated as “enrolled” or if they were “duplicate” based on the fingerprint and would require further review. These are shown in Table 3.

Table 4 shows further review of duplicate fingerprints matched by sex and date of birth, and re-categorized into perfect duplicate, imperfect duplicate, and uncategorized (blanks). Perfect duplicates were records that matched when fingerprints, sex, and date of birth were compared; imperfect duplicates did not match with either sex or date of birth. Of the 162,120 duplicates found, 9591 (5.92%) were perfect duplicates, 18,854 (11.63%) were imperfect duplicates, and 133,675 (82.45%) were uncategorized (Null).

**Table 2.** Fingerprints processed in the ABIS (automated biometric identification system) MegaMatcher from the Nigeria National Data Repository data by June 2024.

Fingerprint status	Fingerprints (N=1,538,971), n	%
Valid data	1,520,187	98.78
No valid data	18,784	1.22

**Table 3.** Outcome of deduplication analysis from ABIS (automated biometric identification system) based on data from the Nigeria National Data Repository by June 2024 (for people living with HIV with valid fingerprints).

Fingerprint status	Client records (N=1,520,187), n	%
Adjudication waiting	93,706	6.16
Duplicate	162,120	10.66
Enrolled	1,264,299	83.17
Exception	62	0

**Table 4.** Manual matching of Nigeria National Data Repository fingerprints data by June 2024 with sex and date of birth to reclassify ABIS (automated biometric identification system) duplicate outcomes.

Duplicate type	Records (N=162,120), n	%
Perfect duplicate	9591	5.92
Imperfect duplicate	18,854	11.63
Null	133,675	82.45

## Discussion

### Principal Results

Our review observed 82.30% of the active client records with valid fingerprint capture were unique, highlighting the potential data quality gap that duplicate records pose to the integrity of a large HIV program. Integrating the fingerprint capture process into routine health care interactions and leveraging the capabilities of the NMRS, NDR, and ABIS MegaMatcher established a comprehensive and efficient system for capturing, storing, and establishing unique system records, thus verifying the uniqueness of clients’ records in the NDR. This methodology helped to identify unique records of clients provided with services, thus preventing double counting. Fingerprint implementation helped to improve the quality of data on the NDR and will help to improve the utility of data from the NDR for programmatic reporting, case-based surveillance, and client-centric monitoring. The number of unique clients receiving treatment is a critical input into the UNAIDS SPECTRUM modeling tool, and better reporting will improve estimation of people living with HIV burden in Nigeria.

A clear resolution protocol that highlights the feedback communication of specific duplicate type or invalid fingerprint records to the health facility or CBO for immediate investigation and resolution is needed to address the different types and patterns of duplicates across locations. Clients with duplicate or invalid fingerprints should be engaged before final determination of validity is reached.

Good-quality data are fundamental to planning public health interventions. Clients may, for reasons of seeking livelihood, migrate from place to place during lifelong HIV treatment without following the standard transfer protocols [21]. These clients could be received as new clients, given new client identification numbers, and reported to the NDR as separate individuals. A pilot study from Malawi was able to determine that approximately 8% of people living with HIV in the study population had more than one client ID in EMR [22]. Fragmented health care systems, client mobility, and inefficient care coordination are some reasons for multiple client registration [23]. Multiple registrations increase the chance of counting clients more than once, thus providing inaccurate reported results. Accurate data are critical for appropriate program planning activities like target setting and budgeting for resources in public health programs.

Fingerprint biometrics can assist with tracking services received by clients, which makes it a useful identifier for longitudinal client monitoring and case-based surveillance [24,25]. Similarly, WHO suggested the use of client biometrics, including fingerprints, for person-centered HIV monitoring and case surveillance [26]. In addition to deduplication of clients using fingerprint records in the NDR for accurate reporting of the number of clients receiving treatment, fingerprints can be used to track sentinel events among HIV cases to inform clinical, programmatic, and public health action.

### Public Health Implications

Biometrics such as fingerprints and the ability to use them to deduplicate clients are critical to program monitoring and planning, effective service linkage, client safety, and person-centered clinical management.

Programs set operational targets each fiscal year. Setting targets depends on factors including target benchmark, resources, and the epidemic gap to be closed. The use of client fingerprints to identify and remove duplicate records is necessary for accurately setting targets and avoiding wastage of resources. Targets help determine resource allocation and budgeting, amplifying the need for good quality data.

Focusing on the scope of sustainability, it will be crucial for countries to have accurate client-level data so they are able to efficiently use funds, make projections, conduct HIV case-based surveillance, and move resources to where they are needed based on the unmet population needs.

### Conclusions

Large-scale deduplication like we have presented was possible because of the NDR, the centralized data warehouse. Integrating fingerprint biometrics into routine HIV program implementation can potentially identify unique client records, prevent double counting, and improving the reliability of records as belonging to unique individuals. The results highlight the potential data quality gap posed by duplicate records, emphasizing the importance of accurate data for programmatic reporting and client-centric monitoring.

### Limitations

The quality of the fingerprint data analysis is dependent on several factors, namely, user, technological (SecuGen, extraction and upload to the NDR, and storage), and the ABIS analytic engine. This paper has not described the methods and algorithms for deduplication of fingerprints in ABIS because ABIS is proprietary.

## Abbreviations

ABIS: automated biometric identification system
CBO: community-based organization
EMR: electronic medical record
NDR: National Data Repository
NMRS: Nigeria Medical Record System
PEPFAR: US President’s Emergency Plan for AIDS Relief
SDK: software development kit
US CDC: US Centers for Disease Control and Prevention
